# Editorial: Workplace violence in healthcare: prevention strategies and mental health consequences

**DOI:** 10.3389/fpsyt.2026.1886605

**Published:** 2026-07-02

**Authors:** Christian Schulz-Quach, C. J. Cabilan, Sanjeev Sockalingam

**Affiliations:** 1 Department of Psychiatry, Temerty Faculty of Medicine, University of Toronto, Toronto, ON, Canada; 2 Workplace Violence Prevention, University Health Network, Toronto, ON, Canada; 3 Mental Health Program, University Health Network, Toronto, ON, Canada; 4 Wilson Centre, University of Toronto, Toronto, ON, Canada; 5 Institute of Health Policy, Management and Evaluation, University of Toronto, Toronto, ON, Canada; 6 Occupational Violence Prevention and Management, Metro South Health, Brisbane, QLD, Australia; 7 Emergency Department, Princess Alexandra Hospital, Brisbane, QLD, Australia; 8 School of Nursing, Midwifery and Social Work, The University of Queensland, Brisbane, QLD, Australia; 9 Centre for Addiction and Mental Health, Toronto, ON, Canada

**Keywords:** burnout, healthcare workers, mental health, moral injury, nursing, occupational psychiatry, occupational violence, prevention

## 1 Introduction

When did we begin to accept that being threatened, struck, or sworn at by the people we care for is simply “part of the job”? Workplace violence (WPV) in healthcare is no longer a marginal occupational hazard — it is a defining feature of the work itself. Verbal abuse, threats, physical assault, and lateral aggression have become so routine that many colleagues describe them in those terms, and that normalization is itself a psychological injury.

Di Martino’s (1) work for ILO/WHO first drew attention to the global scale of WPV. A more recent meta-analysis of 253 studies with 330,000 participants indicates that not much has changed — 6 in 10 healthcare workers are still exposed to any type of WPV from patients and visitors (2). Behind the numbers are people living with the negative impacts — sleep disruption, intrusive memories, depression, moral injury, and, for some, suicidal thoughts into their next shift — a pattern that prospective and longitudinal evidence has now linked consistently to both psychological and physical workplace violence (3).

This Research Topic was conceived to push the conversation beyond prevalence reporting and toward the mental health consequences of WPV and the prevention strategies that might meaningfully address them. The eleven contributions assembled here, drawn from China, India, Italy, Switzerland, and Uganda, offer a coherent picture: WPV is a structural occupational hazard whose psychological sequelae propagate through individuals, teams, and the entire workforce. Read together, they make an argument we believe the field is ready to hear — prevention is much more than a safety and security measure; it is also mental health care.

## 2 Prevalence is established

The contributions align with longstanding data that WPV occurs across different health settings and across all levels of the health workforce.

In India, Vyas et al. reported that 75% of doctors in public and private hospitals experience verbal abuse from patients’ relatives. In China, Liu et al surveyed a large cohort of nurses from 90 hospitals in Sichuan Province; their data showed that 18.4% of nurses had experienced WPV in the previous year, and 34% reported witnessing colleagues being subjected to it. Wang et al. reported a higher WPV prevalence of 69.9% among nurses from secondary and tertiary hospitals in Lanzhou. Further, Zhang et al. found that 85% of emergency nurses had experienced WPV in the previous year.

In many studies, prevalence of WPV was measured from a staff safety perspective. In Italy, Russotto et al. offered an interesting lens — WPV as both a staff and patient safety issue. In their study they documented more than 4,400 patient safety incidents in twelve months, dominated by verbal aggression, physical assault, and death threats.

These studies underscore the scale of WPV across diverse contexts and reframe injuries from WPV as both direct and indirect (vicarious). Additional prevalence studies are not needed. Prevalence, on its own, has stopped being informative; the question worth asking now is what WPV does to those who survive it.

## 3 From incident to injury: the psychological pathway

The consequences span the full spectrum of psychiatric morbidity. Zhang et al. showed psychological violence was the strongest independent predictor of sleep disorders, with HPA-axis dysregulation and rumination as plausible mechanisms. Wang et al. demonstrated that WPV-exposed nurses scored significantly worse across every dimension of occupational wellbeing — physical and mental health, value realization, social support, and work environment.

At the most severe end of the spectrum, Soravia et al. tested a serial mediation model in 88 Swiss rescue workers, including hospital emergency and psychiatric staff. Work-related trauma did not predict suicidal ideation directly; it operated through posttraumatic stress and depressive symptoms, with the model explaining 69% of variance in suicidal thinking. How should we read the distance between a violent encounter and a suicidal crisis? Not as a single event, but as an untreated psychiatric trajectory — in which each step is an opportunity for intervention, and a marker of failure when missed.

Two contributions extend this picture into clinical training and should give educators pause. Zhong et al. found that nursing students’ exposure to WPV was negatively associated with professional commitment. Liang et al., in a separate cohort of nursing students, showed that WPV negatively affected psychological capital and professional identity. What are we asking of future health workers when violence during training does not simply traumatize them but reshapes the professional self that emerges from it?

## 4 Dents on organizational culture

Several contributions situate exposure and harms from WPV within organizational dynamics, with consequences that reach beyond the affected individual into recruitment, retention, and team functioning.

The training-phase findings of Zhong et al. and Liang et al. carry a workforce-pipeline signal: when WPV erodes professional commitment and identity during clinical training, the downstream effect is on who chooses to remain in healthcare — a recruitment problem that begins long before nurses and physicians enter the workforce.

Liu et al., in the first study of psychiatric nurses to model these dynamics directly, provide evidence linking WPV to turnover intention through ward atmosphere and social distance as mediators. WPV worsens perceptions of the work environment and of relationships with colleagues and patients, which in turn drives psychiatric nurses to consider leaving — confirming that the psychological climate of a unit is itself an exposure variable.

Tumusiime et al., in a Ugandan referral hospital, extend these findings to resource-constrained settings, where 64.5% of nurses reported occupational stress and continuous professional development emerged as protective.

Liu et al. found that transformational leadership by head nurses increased engagement, with horizontal violence accounting for 40% of the mediating effect. Where leadership is weaker, lateral aggression flourishes and engagement collapses.

Together, these papers make visible what has been hiding in plain sight. WPV is not only an interaction between aggressor and target; it is a property of the system in which both are embedded. Why, then, do so many of our prevention programs still treat it as a series of individual encounters?

## 5 Implications: prevention as a mental health intervention

The contributions in this Topic orient prevention toward psychiatric outcomes rather than incident counts. The Soravia pathway from trauma to PTSD to depression to suicidal ideation invites every WPV program to plan for the early identification and treatment of post-event psychiatric morbidity, not only post-incident debriefing. Within this orientation, four prevention pillars structure the work ahead, each reinforced by the contributions to this Topic and by broader review evidence (4).

### 5.1 Prevention through staff education, training, and recovery

Training-phase exposure demands particular attention because it shapes the workforce that emerges. Capacity-building tele-education models such as ECHO Ontario Mental Health from Canada, which has built primary-care mental health capacity through hub-and-spoke virtual learning (5) and has been shown to create a safe space for reflection on complex team dynamics, uncertainty, and productive struggle relevant to WPV (6), offer a scalable architecture for equipping the workforce to recognize, treat, and refer post-WPV psychiatric morbidity. The Trauma-Informed De-escalation for Safety (TIDES) program at the Centre for Addiction and Mental Health (CAMH) in Toronto, designed with an explicit health-equity and inclusion framework (7), illustrates how this architecture can be operationalized for frontline staff with explicit attention to post-incident recovery.

### 5.2 Prevention through environmental design

The physical environment is a modifiable risk factor too often overlooked in WPV programs. A recent scoping review of emergency department environmental interventions identified four design domains — preventing harm from weapons, controlling physical access, observation and awareness, and patient comfort — that frontline staff perceive as material to their safety (8). Crime Prevention Through Environmental Design (CPTED) principles offer a structured way to embed these features into healthcare facility planning.

### 5.3 Prevention through patient risk assessment and coordinated team response

Practical structured tools matter at the point of care. Cabilan and colleagues’ three-domain Queensland Occupational Violence Patient Risk Assessment from Australia, embedded in the emergency department workflow, was associated with progressive declines in reported violent incidents and offers a replicable point-of-care prevention mechanism (9). Systematic review evidence confirms that validated risk-assessment tools, combined with coordinated team response models (e.g., Code White behavioural emergency response), are essential elements of effective WPV reduction (4).

### 5.4 Prevention through inter-agency collaboration and systems integration

WPV is a systems problem rather than a sequence of discrete safety interventions, and no single institution can resolve it in isolation. The WHO/ILO Framework Guidelines for Addressing Workplace Violence in the Health Sector (1) set the global precedent for cross-sector collaboration across health, labour, policing, and community services. A recent Canadian quality-improvement initiative at the University Health Network, guided by the SEIPS 3.0 framework, demonstrates how a trauma-informed twelve-step approach can integrate reporting infrastructure, leadership engagement, environmental design, staff support, and a culture of mutual respect into one prevention architecture (10) — a translational template for operationalizing the signals in this Topic across institutional and inter-agency layers.

Our contributors invite us to see WPV for what it has long been — a sustained occupational exposure with real mental health consequences for the healthcare workforce, and a system problem that requires system-level redesign. We hope readers will join us in the task ahead: building prevention architectures that treat the safety of healthcare workers and the safety of their patients as one and the same.
